# Feature amplified voting algorithm for functional analysis of protein superfamily

**DOI:** 10.1186/1471-2164-11-S3-S14

**Published:** 2010-12-01

**Authors:** Che-Lun Hung, Chihan Lee, Chun-Yuan Lin, Chih-Hung Chang, Yeh-Ching Chung, Chuan Yi Tang

**Affiliations:** 1 Department of Computer Science, National Tsing Hua University, 101, Section 2 Kuang Fu Road, Hsinchu, Taiwan 300; 2 Macronix International Co., Ltd., 16, Li-Hsin Road, Science Park, Hsinchu, Taiwan 300; 3 Department of Computer Science and Information Engineering, Chang Gung University, 259 Wen Hwa 1st Road, Kwei Shan, Taoyuan 333, Taiwan.

## Abstract

**Background:**

Identifying the regions associated with protein function is a singularly important task in the post-genomic era. Biological studies often identify functional enzyme residues by amino acid sequences, particularly when related structural information is unavailable. In some cases of protein superfamilies, functional residues are difficult to detect by current alignment tools or evolutionary strategies when phylogenetic relationships do not parallel their protein functions. The solution proposed in this study is Feature Amplified Voting Algorithm with Three-profile alignment (FAVAT). The core concept of FAVAT is to reveal the desired features of a target enzyme or protein by voting on three different property groups aligned by three-profile alignment method. Functional residues of a target protein can then be retrieved by FAVAT analysis. In this study, the amidohydrolase superfamily was an interesting case for verifying the proposed approach because it contains divergent enzymes and proteins.

**Results:**

The FAVAT was used to identify critical residues of mammalian imidase, a member of the amidohydrolase superfamily. Members of this superfamily were first classified by their functional properties and sources of original organisms. After FAVAT analysis, candidate residues were identified and compared to a bacterial hydantoinase in which the crystal structure (1GKQ) has been fully elucidated. One modified lysine, three histidines and one aspartate were found to participate in the coordination of metal ions in the active site. The FAVAT analysis also redressed the misrecognition of metal coordinator Asp57 by the multiple sequence alignment (MSA) method. Several other amino acid residues known to be related to the function or structure of mammalian imidase were also identified.

**Conclusions:**

The FAVAT is shown to predict functionally important amino acids in amidohydrolase superfamily. This strategy effectively identifies functionally important residues by analyzing the discrepancy between the sequence and functional properties of related proteins in a superfamily, and it should be applicable to other protein families.

## Background

(The software is freely available for download from reference [1]).

Retrieving useful functional/structural information from a set of amino acid sequences is essential in experimental biological studies. Desired information is often obtainable by analyzing the sequence conservations, functional correlations and related structures that belong to a protein/enzyme family or superfamily. An enzyme superfamily is defined as a group of proteins that share the same structural scaffold and that undergo fundamentally similar chemical reactions [2]. Earlier studies [3-5] adopted various pair-wise alignment and multiple sequence alignment (MSA) methods to detect the conserved residues that reveal functional roles in a set of sequences. Classical sequence comparison tools such as FASTA [6], BLAST [3], CLUSTALW [7], T-COFFEE [8] and MUSCLE [9] can detect similarities in aligned sequences and identify the conserved positions. These positions are essential for further functional analysis. Hierarchical analysis [10-12] is often used to select the most desirable pattern of an alignment. Some protein groups with dissimilar sequences but substantial structural fold similarity (hereinafter referred to as remote homologues) have similar or related biochemical functions [13]. These proteins can be classified into the same superfamily according to their biological properties. Due to their low overall similarity, using alignment methods alone may not reveal the amino acid residues that reflect their physicochemical properties.

In addition to alignment methods, the most common strategy for predicting functional residues from sequences is motif-based sequence analysis [14-17]. However, the motif-based approach often obtains excessive false positives, which limits its use for analyzing a protein superfamily. Phylogenomic techniques such as the evolutionary trace method of identifying functionally important residues [18] use evolutionary information to improve accuracy and are particularly useful for large-scale analyses. This method automatically relates the results back to a given structure and identifies key features structurally clustered around substrate and dimmer interfaces [19-22]. This tool is useful for analyzing protein or enzyme superfamilies and for extracting functional information from enzyme families or superfamilies when the phylogenetic tree or dendrogram approximates a functional distribution.

This study employs a voting concept to search for functional key residues in an enzyme superfamily. Voting or voting-like concepts are widely used in computing algorithms for various purposes. In computational biological applications, voting concepts are often integrated with neural networks for protein clustering and structure prediction [23]. Some theoretical analyses [24-29] indicate that comparing three sequences is better than comparing two sequences because it increases the alignment power needed to distinguish significant matches. Likewise, aligning three groups provides more information than aligning two groups does. Therefore, we developed a Feature Amplified Voting Algorithm with Three-profile alignment (FAVAT) according to the observed sequence similarity and biochemical properties of proteins in the amidohydrolase superfamily. The FAVAT identifies the key residues by calculating a score for each residue in a rat imidase. The functional residues of a rat imidase were identified and further confirmed by experimental references and available structural information.

## Results and discussion

### Case study: Imidase-related proteins in amidohydrolase superfamily

In this study, rat imidase was the target sequence, and DRPs (Group II proteins) were classified into ~A proteins. Bacterial hydantoinases (Group III enzymes) were classified as A proteins. Although Dihydroorotase, allantoinase and other amidohydrolases (Group IV) were also classified as A proteins, they differ from the Group II enzymes in their functional correlation to target sequence (rat imidase). Following the above classification, the clustered sequences were subjected to FAVAT analysis, and two sets of scores were obtained for each residue of the target sequence. In the experiments, Groups II, III and IV were aligned using the MUSCLE tool adopted by the National Center for Biotechnology Information (NCBI) for protein database alignment.

### Voting scores of imidase by FAVAT analysis

**Figure 1:**
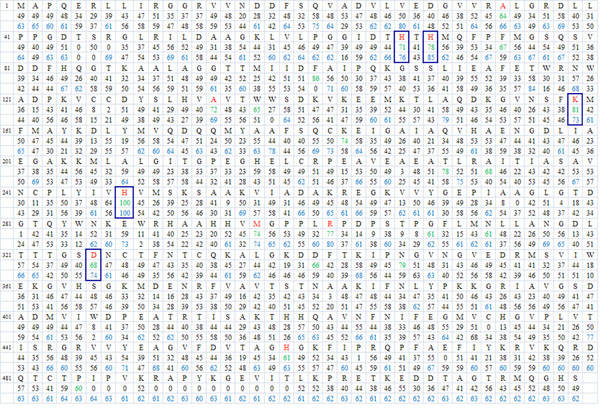
**Total voting scores (T-scores) of rat imidase by FAVAT analysis** The scores were obtained by voting algorithm as described in Methods. The first row is the rat imidase sequence. The scores in the second and third rows are accumulated V-scores (T-score) for the Group II-Group III and Group II-Group IV votes, respectively. Amino acid residues are marked in red if both votes resulted in T-scores over 60. The T-scores over 60 for Group II-Group III and Group II-Group IV votes appear in green and blue, respectively. The boxed residues indicate the residues corresponding to metal coordination in bacterial hydantoinase (1GKQ).

Figure 1 shows the FAVAT analysis results. The T-score suggests the importance of each residue of the target sequence (rat imidase) after accumulating the total voting scores (V-scores), each V-score is calculated from the target sequence, an A protein sequence and a ~A protein sequence. For each rat imidase residue, two sets of T-scores were obtained from the Group II-Group III votes and the Group II-Group IV votes. Ten amino acids (Ala34, His67, His69, Ala134, Lys159, His248, Met297, Arg302, Asp326 and His459) were further analyzed after merging the two sets of the higher T-scores over 60. Table 1 summarizes the amino acid residues selected by FAVAT and MSA analyses and their corresponding locations in two proteins with known crystal structures [PDB:1GKQ, PDB:1KCX].

**Table 1:** Functional annotations of the residues in rat imidase selected by FAVAT

FAVAT score ranking	FAVAT selected residues	Corresponding residues in 1GKQ^1^	Corresponding residues in 1KCX^1^	MSA predicted residues^2^	Functional annotation base on 1GKQ
1	**His248**	**His239**	**Lys254**	**His239**	**Metal coordinate**
2	**His69**	**His61**	**Tyr75**	**His61**	**Metal coordinate**
3	Arg302	Arg292	Ser308		Conformation
4	**Lys159**	**Lys150 (Kcx)**	**Gln165**		**Metal coordinate**
5	Met297	Met287	Thr303		Conformation
6	**His67**	**His59**	**Asn73**	**His59**	**Metal coordinate**
7	**Asp326**	**Asp315**	**Gly332**		**Metal coordinate**
8	Ala134	Ala126	Asp139		**Secondary structure core residue**
9	Ala34	Arg30	Gln44		Quaternary structure
10	His459	Trp448	Met465		Quaternary structure

^1^GKQ and 1KCX are the structure IDs of D-hydantoinase and collapsin response mediator proteins (CRMPs), respectively.

^2^MSA result according to the manuscript [66].

### Comparison of FAVAT and MSA results

**Figure 2:**
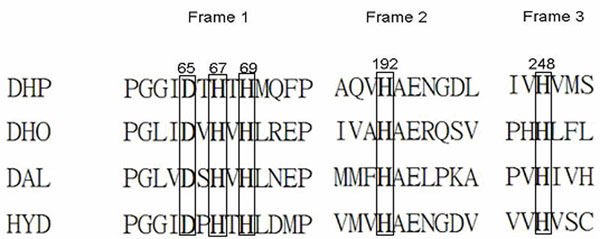
**Results of MSA analysis** A multiple sequence alignment of imidase (DHP), dihydroorotase (DHO), allantoinase (DAL) and D-hydantoinase (HYD) was performed using CLUSTALW. The conserved amino acid residues among the four enzymes are shown in bold type and enclosed in rectangles.

Biologists often use MSA to conjecture important residues of proteins or enzymes of interest among their related sequences. Figure 2 shows the MSA fragments of rat imidase, hamster dihydroorotase domain, yeast allantoinase and Bacillus sp. D-hydantoinase. The MSA analyses revealed that one aspartate and four histidines were highly conserved. The results in Fig. 2 are consistent with data published previously [30-32].

**Figure 3:**
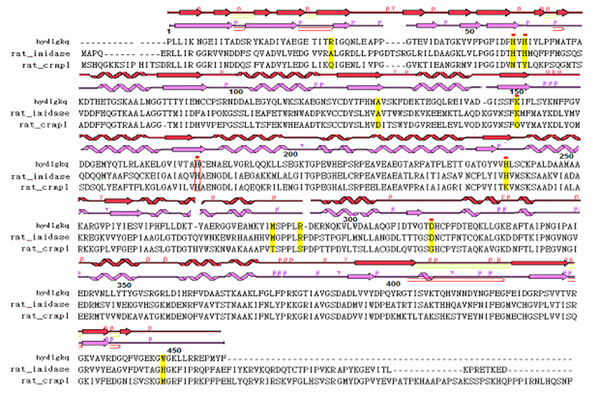
**Corresponding locations of FAVAT-selected residues in the wiring diagrams of 1KCX and 1GKQ** The secondary structures are those of CRMP-1 (1KCX, red font) and D-hydantoinase (1GKQ, blue font), respectively. The sequences are for D-hydantoinase (hydlgkq), rat imidase (rat_imidase) and rat CRMP1 protein (rat_crmp1). The top ten residues obtained by FAVAT analysis are highlighted in yellow. Residue His183, which was not among the top ten residues selected by FAVAT, is boxed in red. Residues indicated by dotted lines were those reported for metal coordination of hydantoinase.

In their studies [30-32], residues Asp57, His59, His61, His183 and His239 were hypothesized to be critical amino acids for the metal coordinators and function of D-hydantoinase of Thermus sp. Notably, the study [33] reported that a crystal structure of hydantoinase (1GKQ), in which a carboxyl-lysine is responsible for metal binding and is important for enzyme activity, was not revealed by MSA analysis. Another residue, Asp57, which was incorrectly identified as a metal coordinator in a previous study that applied MSA method as the corresponding residue in 1GKQ, revealed no involvement in metal coordination. Table 1 shows that, in the current study, FAVAT successfully identified all known important residues in rat imidase. The Lys159, His67, His69, His248 and Asp326, which correspond to Lys150, His59, His61, His239 and Asp315 in hydantoinase (1GKQ), are metal ion coordinators (boxed amino acids in Fig. 3).

### The corresponding locations of FAVAT-selected residues in 1GKQ and 1KCX

The possible functions of imidase amino acids selected by FAVAT were further analyzed using 1GKQ and 1KCX, which are known structures of imidase related proteins. The former is the crystal structure of a D-hydantoinase that represents an A protein (Group III) in FAVAT analysis. The latter is the crystal structure of a dihydropyrimidinase-related protein (CRMP1) that represents a ~A protein (Group II) in FAVAT analysis. Figure 3 shows their corresponding sequences and secondary structures. The similar β/α core structures were observed in the wiring diagrams of 1GKQ and 1KCX. The significant difference in these structures is that 1GKQ forms a typical (β/α)_8_ domain, but 1KCX does not. The FAVAT-selected amino acids may reflect both the structure feature and metal requirement that are responsible for the different functions of the A and ~A proteins. The corresponding locations of Ala34 and His459 in 1GKQ (Arg30 and Trp448 in the N-terminal and C-terminal β-Sheet, respectively) and in 1KCX (Gln44 and Met465 in the N-terminal and C-terminal β-Sheet, respectively) were domains in which they interact with another monomer to form a quaternary structure in both hydantoinase and CRMP1 [34,35]. Residues His67, His69, Ala134, Lys159, His248 and Asp326 (His59, His61, Ala126, Kcx150, His239 and Asp315 in 1GKQ; His73, Tyr75, Asp139, Gln165, Lys254 and Gly332 in 1KCX) are located in the β/α core region.

**Figure 4:**
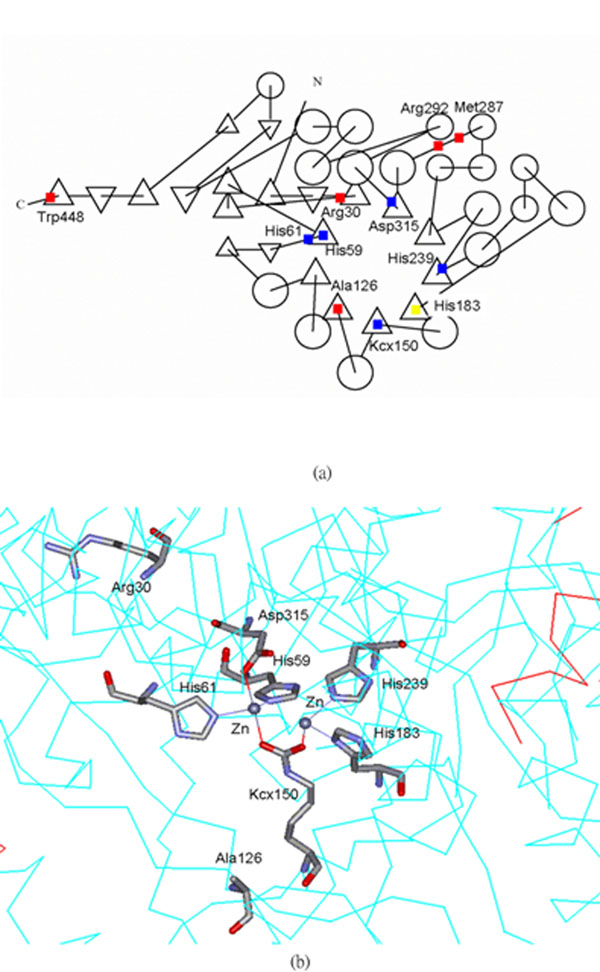
**Corresponding locations of amino acid residues identified by FAVAT in 1GKQ** (a) The top ten residues selected by FAVAT on the topology of 1GKQ. Carboxylated lysine residue (Kcx150), His59, His61, His239 and Asp315 that were involved in metal coordination are indicated by blue font. Residue His183 (yellow font) was not identified in this study. Other selected residues are indicated by red font. (b) The metal binding site of 1GKQ. The corresponding amino acids identified by FAVAT are highlighted.

Figure 4a is a topological view of the corresponding FAVAT-selected amino acids in 1GKQ. Thermus sp. D-hydantoinase (1GKQ) contains two divalent metal ions in active site (Fig. 4b). The central binuclear zinc center is bridged by the carboxylated lysine residue (Kcx150) and a hydroxide ion. Residues His59, His61, His183, His239 and Asp315 correspond to the active site zinc ion (Fig. 4b). The corresponding residues were conserved in other members of the amidohydrolase superfamily [36,37]. However, in CRMP1, four of the five corresponding residues are diverse [38]. These residues were all identified by FAVAT analysis. Other candidates recognized by FAVAT analysis, Ala134, Arg302, Ala34, Met297 and His459, were also found to reside in positions critical to protein function. The corresponding residue of Ala134 in 1GKQ (Ala126), was located near the active site (Fig. 4b). Residues Arg302 and Met297 (Arg292 and Met287 in 1GKQ, respectively) were located at the helix-loop domain outside the (β/α)_8_ catalytic domain. This implies that they may be important for maintaining structure or stabilizing the protein conformation (Fig. 4a). These preliminary findings merit further detailed study.

Although the metal coordinators of imide-hydrolyzing enzymes in this case study were dispersed sequentially, almost all the known metal coordinators in 1GKQ were identified by FAVAT except His 183 (His 192 for rat imidase). This residue is conserved in CRMP1 but lacks metal and amidohydrolytic activity. The role of this histidine needs further study. The major difference between bacterial hydantoinase and mammalian imidase is their metal content. The former contains two metal ions while the later contains only one metal ion [39,40]. Fewer metal coordinators may be needed for mammalian imidase, and residue His 192 may not be required as a coordinator of metal ions in rat imidase. A mammalian imidase was crystallized recently [41]. The difference between mammalian imidase and non-mammalian imidase is expected to be clarified in the near future.

## Conclusions

The FAVAT was developed to predict functionally important amino acids in mammalian imidase. A T-score was given to each residue of the target enzyme by analyzing imidase-related proteins in the amidohydrolase superfamily on the basis of their sequence-function relationships. Of the ten top T-score amino acids selected, six (His67, His69, Lys159, His192, His248 and Asp326) corresponded to metal coordination in D-hydantoinase. The other four amino acids corresponded with positions that were structurally important for forming quaternary structures and secondary structures in 1GKQ. Residue Asp57, which was misrecognized as a metal coordinator in previous MSA analyses, was correctly recognized by FAVAT. This study showed that analyzing the discrepancy between the sequence and functional properties of related proteins in a superfamily is an effective method of identifying functionally important residues. This strategy should be applicable to other protein families, and the authors expect to employ this strategy for analyzing critical residues of viruses in future works.

## Methods

### Imidase and sequence clustering in the amidohydrolase superfamily

Hydantoinase activity was first reported in plants and animals [42,43] to hydrolyze hydantoin derivatives that are not known as physiological metabolites. This enzymatic activity is useful for preparing optically pure amino acids that are precursors for various antibiotics [44]. Due to its industrial application, several hydantoinases have been studied and purified from microorganisms [45,46]. A dihydropyrimidinase (5, 6-dihydropyrimidine amidohydrolase) partially purified from animal livers was shown to hydrolyze the physiological substrate dihydropyrimidine [47]. A detailed study of a homogenous imide-hydrolyzing enzyme, imidase, which was purified from rat, pig or fish livers [48-51], revealed that it catalyzes a wide spectrum of substrates, including dihydropyrimidines, hydantoins and other imides [52]. Despite the substrate spectra of hydantoinase highly similar to imidase, these imide-hydrolyzing enzymes from bacterial and mammalian sources reportedly have relatively low sequence similarity. Some mammals, flies and C. elegans, reveal proteins with high sequence similarity to dihydropyrimidinase (or imidase). These dihydropyrimidinase-related proteins (DRPs) may be involved in cancer and neuron cells development, but possess no imidase activity [53-55]. Additionally, other enzymes revealed by the studies in evolution of the metabolic pathway are also known to use mechanisms similar to those observed in imidase [56,57]. These enzymes include dihydroorotase, allantoinase, urease and amidohydrolases, which originate in mammals, plants and fungi [58]. All use distinct substrates that contain similar imide functional groups.

All of the above enzymes can be classified into the amidohydrolase superfamily according to their properties and structures [59]. In this superfamily, some proteins have similar sequences but divergent functions whereas others have similar functions but low sequence similarity. This phenomenon strongly suggests that only a few critical amino acid residues in this superfamily are needed for specific protein functions. Proteins in the amidohydrolase superfamily can be grouped according to their sequence similarity and biochemical properties, and an effective strategy for analyzing these proteins may yield valuable information.

A string search containing the rat imidase sequence (Accession No.: NP_113893) yielded 156 protein sequences of amidohydrolase superfamily were obtained from the PIR database [60]. According to their sequence similarity and functional properties, sequences were further clustered into the following five groups (Table 2): I. imidase (imide-hydrolyzing enzyme from mammal); II. sequence-related proteins (dihydropyrimidinase-related proteins with 50% or higher sequence similarity to mammalian imidase but without imidase activity); III. functionally identical enzymes (hydantoinase, or the imide-hydrolyzing enzyme from bacteria with 30-40% sequence similarity to mammalian imidase); IV. functionally-related enzymes (dihydroorotase, allantoinase, urease and amidohydrolase with 25-48% sequence similarity to mammalian imidase); and V. putative sequences (gene products with unknown function) with 30% or higher sequence similarity to mammalian imidase.

**Table 2:** Grouping of imidase related proteins^1^

Group	Member number	Sequence identity	Imidase Activity
I. Imidase (target enzyme)	5	98-100%	Yes
II. Sequence related proteins^2^	43	50-80%	No
III. Functionally identical enzymes^3^	16	30-40%	Yes
IV. Functionally related enzymes^4^	63	25-48%	Yes
V. Putative proteins^5^	29	30-60%	Unknown

^1^Proteins are grouped according to properties related to the target enzyme (rat imidase)

^2^Sequence-related proteins, referred to as dihydropyrimidinase related proteins (DRPs), with no imidase activity.

^3^Functionally identical enzymes, referred to as bacterial imide-hydrolyzing enzyme or hydantoinase, in which substrate spectrums were virtually identical to that of the target enzyme.

^4^Functionally-related enzymes, including dihydroorotase, allantoinase, and urease. Each enzyme catalyzes its own distinct substrate that contains an imide functional group.

^5^ Putative proteins are referred to as gene products with unknown function.

### Observation and assumption

**Figure 5:**
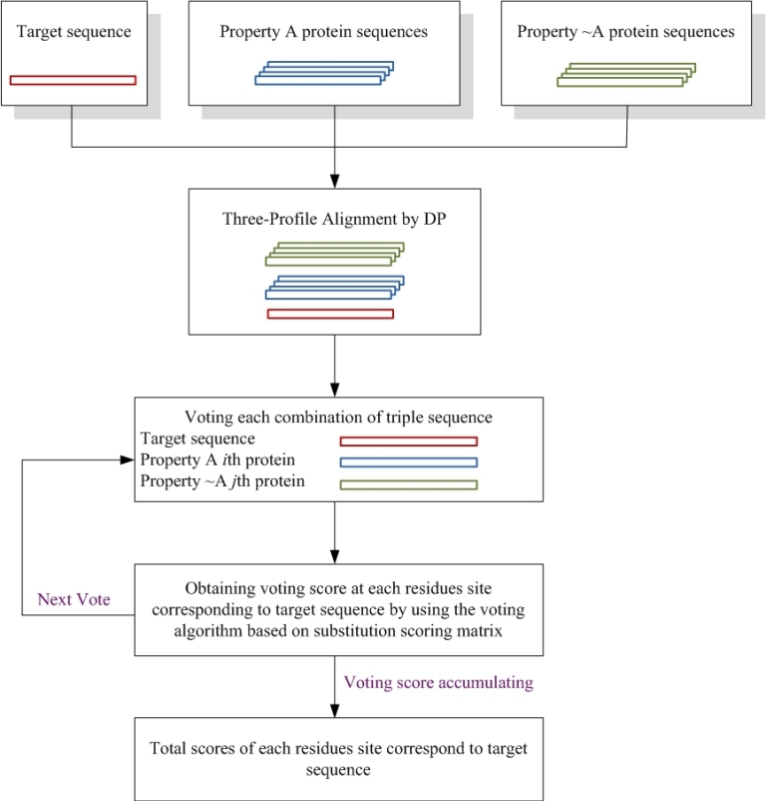
**Flowchart of FAVAT** In this study, rat imidase was first aligned simultaneously to A proteins and ~A proteins to obtain voting scores (V-scores) by voting algorithm based on BLOSUM62 substitution matrix. The V-scores for each vote were accumulated at the corresponding residues in the target sequence and used to obtain a total score (T-score).

Table 2 shows the significant findings of the comparisons of sequence identity and functional properties of the imidase-related proteins in the amidohydrolase superfamily. Sequence-related proteins (Group II) had no imidase activity even though the overall similarity of sequences in this group was higher than 50% [61]. This phenomenon implicated that key amino acid residues for imidase activity have been altered or removed from Group II proteins. In contrast, functionally identical and functionally related enzymes (Group III and Group IV, respectively) had lower sequence identity, but they basically catalyzed the same reaction. For these enzymes, few conserved residues should be needed to provide a similar imidase function. The above observation implies a principle for classifying the sequence or the functional divergent enzymes in a superfamily, which may help to develop a feature amplified voting algorithm for identifying key residues in a target protein. Biologists generally select a sequence of interest as a target and perform BLAST analysis to recover related protein sequences. By definition, a specific function or property (*e.g.,* substrate specificity) of a sequence of interest can be used to cluster the related sequences into different groups. For example, if property A is used to classify these sequences, sequences with property A should be classified into group A. Otherwise, the sequence should be classified into group ~A. Based on the classification of these three groups, a critical assumption can be made. When the target sequence, the A sequence and the ~A sequence belong to a protein superfamily, the conserved residues of the target sequence and the A sequence should correlate to property A. However, the non-conserved residues of the target sequence and ~A sequence should also reflect the negative correlation in property A. The intersection of the residues provides important functional clues. Thus, important residues can be found by comparing these three property groups. Therefore, the three-profile alignment method was developed to optimize the alignment of three groups so that key residues are then revealed by voting algorithm. Figure 5 shows the FAVAT flowchart that was developed to retrieve useful information for these distinct groups. Analysis of the imidase-related sequences in Table 2 indicated that, within this superfamily, the degree of sequence similarity did not necessarily reflect the similarity in biochemical properties. This provides a good opportunity to develop a novel method for extracting functional residues of a target enzyme. In this work, FAVAT was used to examine each residue in mammalian imidase by comparing other sequences in the amidohydrolase superfamily. Given the limited structural information about mammalian imidase, the analytical results of this study should provide important clues for enzymologists to perform further in-depth biochemical analyses of these results.

### Algorithm

The FAVAT was performed in two steps. The first step was to align the target enzyme, functionally identical enzymes (A proteins) and sequence-related proteins (~A proteins) using three-profile alignment. The three-profile alignment algorithm, which is based on the dynamic programming three-way alignment approach [62,63], was designed to align three profiles in a space. As in the FAVAT pre-process, each profile can be generated by multiple sequence alignment tools such as T-COFFEE, HMMER [64] and MUSCLE.

**Figure 6:**
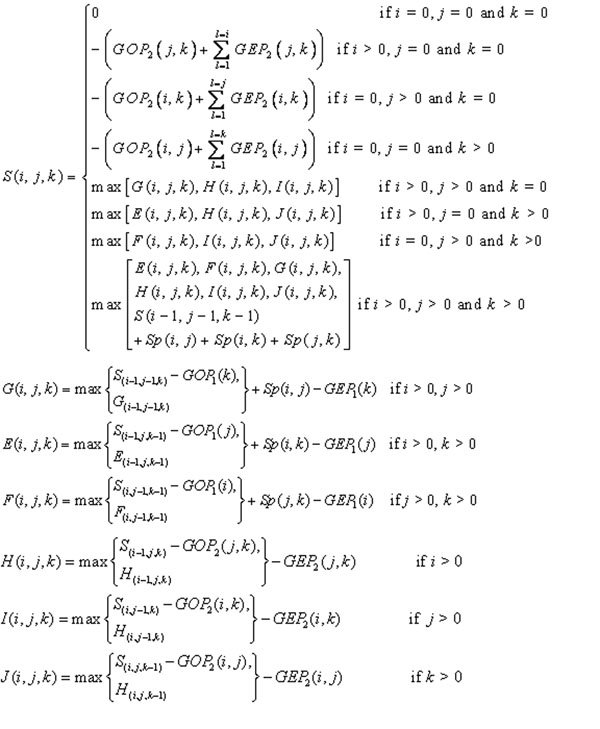
**Dynamic programming recursions for three-profile alignment with affine gap costs** Scores for the alignment of two residue positions are denoted by *Sp(α,* β). The *GOP* and *GEP* are gap opening and gap extension penalties, respectively. In affine gap costs, *GOP* and *GEP* are fixed values at any residue position.

Let *P*_1_, *P*_2_ and *P*_3_ be three profiles, and *P*_1*i*_, *P*_2*j*_ and *P*_3*k*_ refer to the *i*th, *j*th and *k*th positions in *P*_1_, *P*_2_ and *P*_3_, respectively, starting from 1. The symbol “-” denotes a “gap” in the alignment. Scores for the alignment of two columns are denoted by *Sp*(α, β). The scoring pair profiles *P*_1_-*P*_2_ are defined as follows:

where *Sp*_12_ is the score at the *i*th and*j*th columns on *P*_1_ and *P*_2_, respectively. The *P*_1_ has *m* sequences, and *P*_2_ has *n* sequences. The *W_a_* and *W_b_* are the sequence weights for sequence *a* in *P*_1_ and sequence *b* in *P*_2_, respectively. The residue at *i*th column for sequence *a* in *P*_1_ is denoted by *r*_1(*a,i*)_. The *M* is the value of the substitute matrix for *r*_1(*a,i*)_ and *r*_2(*b,j*)_. Many substitution matrices, such as BLOSUM, have been proposed to improve alignment accuracy [65]. Similarly, the definitions of scoring pair profiles *P*_1_-*P*_3_ and *P*_2_-*P*_3_ are similar to those of pair profile *P*_1_-*P*_2_. Gap penalties are determined by gap opening (*GOP*) and gap extension (*GEP*) scores. The best score of the alignments with prefixes *P*_1*i*_, *P*_2*j*_ and *P*_3*k*_ is denoted by S(*i*, *j*, *k*) if the residues (*P*_1*i*_, *P*_2*j*_, *P*_3*k*_) are aligned; *G*(*i*, *j*, *k*) is the best score given that (*P*_1*i*_, *P*_2*j*_, -) is the last column of the partial alignment, and H(*i*, *j*, *k*) is the best score given that the last column is of the form (*P*_1*i*_, -, -). *E*(*i*, *j*, *k*), *F*(*i*, *j*, *k*), *I*(*i*, *j*, *k*) and *J*(*i*, *j*, *k*) are defined analogously. These quantities clearly satisfy the recursions summarized in Fig. 6.

**Figure 7:**
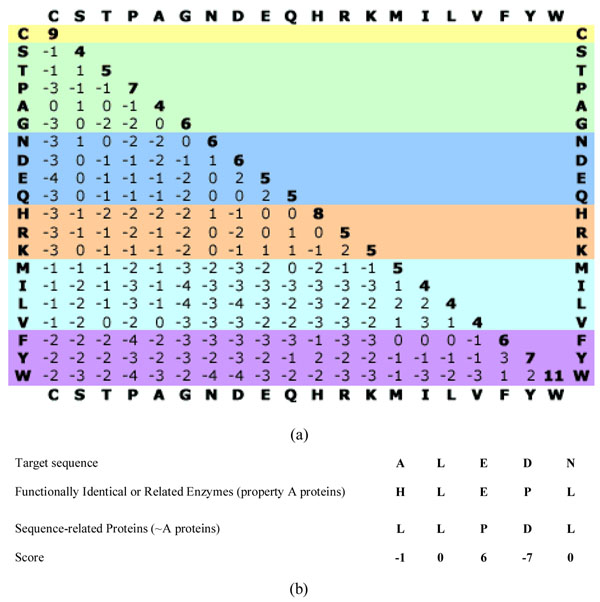
**Score setting and scoring example** (a) Substitution matrix for BLOSUM62. In this study, the BLOSUM 62 substitution matrix was used to obtain V-scores for the voting algorithm. (b) Example of FAVAT scoring. When three-profile alignment was performed in the target sequence, the voting scores for A proteins and ~A proteins corresponded with the BLOSUM62 substitution matrix. For example, residues for target sequence, an A protein and a ~A protein were A, H and L, respectively. The V-score is -2 (A to H in BLOSUM62) - (-1) (A to L in BLOSUM62) = -1.

**Figure 8:**
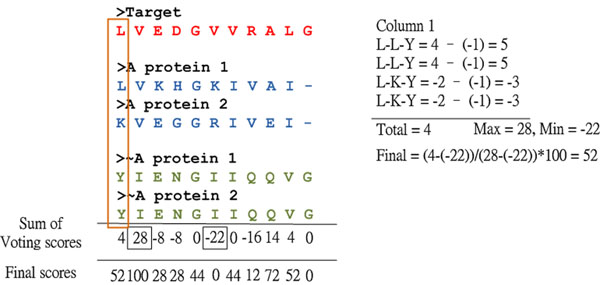
**FAVAT scoring example** For the first position of the target sequence, four V-scores were calculated for L-L-Y, L-LY, L-K-Y and L-K-Y, respectively. The T-score at each position is the sum of four V-scores, and the T-score (total score) for the first position of the target sequence is 4. After the normalization function, the final T-score is 52. When any gap existed at the position in one of three groups such as column 11, the T-score for this position is zero.

The next step after the alignment is to determine whether amino acid residues critical for imidase activity exist in target and A proteins but are absent in ~A proteins. In the second step, a voting score (V-score) is given based on the previous assumption, and the V-scores are then summed in each comparison. In this step, a substitution matrix (BLOSUM62) is used to give V-score when each sequence of property A and ~A is compared to the target sequence. The V-score is calculated as follows:

*V*_*k*(*a,b*)_ = *M*[*t_k_*, *A*_(*a,k*)_] – *M*[*t_k_*, ~*A*_(*b,k*)_],

where *V*_*k*(*a,b*)_ is the V-score at the *k*th residues on target sequence, sequence *a* in A proteins and sequence *b* in ~A proteins. The A and ~A proteins have *m* and *n* sequences, respectively. The *t_k_* is the *k*th residue of the target sequence. The *A*(*a,k*) and ~*A*(*b,k*) are the *k*th residues on sequence *a* in A proteins and on sequence *b* in ~A proteins, respectively. The *M*[*t_k_*, *A*_(*a,k*)_] is the value of the substitution matrix for *t_k_* and *A*_(*a,k*)_. Figure 7 shows the V-score calculation. The BLOSUM62 substitution matrix is generally used for protein or nucleic acid sequence alignment. In the proposed algorithm, each V-score is given and accumulated to a total score (T-Score) until all sequences of property A and ~A are compared. The T-Score is calculated as follows:

where *T_k_* is the T-Score at the *k*th residue on target sequence. The T-Score at each residue position is obtained by adding *m × n* V-scores. The normalization function is used to transform all T-scores into a range from 0 to 100 in FAVAT. The normalization function is as follows:

where *Max*(*T*) and *Min*(*T*) are the maximum and minimum scores of all T-scores, respectively. The details of the FAVAT algorithm are shown below, and Fig. 8 presents an example of V-Score and T-Score calculations using FAVAT.

***Algorithm FAVAT*** (*t, P, Q*);

**Input:** Target sequence *t*, a set of proteins *P* without property *A*, a set of proteins *Q* with property *A*. *P* has *p* sequences and *Q* has *q* sequences.

**Ouput:** The scores correspond to the residues of *t* (high T-scores indicate potentially critical residues)

*Begin*

1 /*Step1: Do three-profile alignment by dynamic programming method among *t*, the proteins *P*, and the proteins *Q*. The length of the resulting alignments is *m_max_*. *t*[*k*] indicates the *k*-th residue of *t*.*/

2 **for***k* <- 1 **to***m_max_***do**

3 **if***t*[k] <> ‘-‘ **then**

4 /*T-score [*k*] indicates the potential importance of the *k*-th residue of *t*. */

5 *T-score*[*k*] <- 0

6 *max* <- -∞

7 *min* <- ∞

8 **for***i* <- 1 **to***p***do**

9 **for***j* <- 1 **to***q***do**

10 /*X and *Y* are used to store the k-th residue of this *i*-th protein in *P* and this *j*-th protein in *Q*, respectively.*/

11 (*X*,*Y*) = (*P*[*i*], *Q*[*j*], *k*-th)

12 /*Step 2: Find *V-score*[*k*] based on the BLOSUM62 substitution matrix.*/

13 *V-score*[*k*]<-BLOSUM62(*t*[*k*], *Y*)

14 *V-score*[*k*]<*-V-score*[*k*] + (-1) × BLOSUM62(*t*[*k*], *X*)

15 *T-score*[*k*] <- *T-score*[*k*] + *V-score*[*k*]

16 *max* <- *MAX* (*max, T-score*[*k*])

17 *min* <- *MIN* (*min, T-score*[*k*])

18 **end if**

19 **for***k* <- 1 **to***m_max_***do**

20 *T-score*[*k*] = (*T-score*[*k*] – *min/max – min*) × 100 /*normalization*/

End

The novel feature of the FAVAT algorithm is its use of the sequence and functional properties among target sequence, ~A proteins and A proteins. When voting for reliable critical residue candidates, three relations are considered: the relation between target sequence and A proteins, the relation between target sequence and ~A proteins and the relation between A proteins and ~A proteins. To accurately identify the key residues, some useful alignment tools with physicochemical properties, such as T-COFFEE and HMMER, can be employed in the FAVAT pre-process to align A and ~A proteins separately (profiles). The appropriate alignments of A and ~A proteins can enhance the accuracy of the resulting alignment to the target sequence, A and ~A proteins by three-profile alignment. The most important residues can then be found accurately from the resulting alignment using FAVAT. The FAVAT algorithm was designed to account for the importance of alignment-based voting skill by V-score function. The time complexity of FAVAT is O(m_*max*_^3^), and m_*max*_ is the length of the resulting alignment by three-profile alignment. To reduce the time complexity of three-profile alignment method, this study developed a parallel version implemented by the MPICH library. The time complexity for the parallel version is O(m_*max*_^3^/*p*), where *p* is the number of processors. After the voting process, the residue candidates obtain high T-scores. The uncritical candidates can be eliminated by advanced research.

## Competing interests

The authors declare that they have no competing interests.

## Authors' contributions

CLH, CL and CYT conceived the research. CLH and CL implemented the program and performed the experiments. CLH, CL and CYL arranged the test data and analyzed the experimental results. CLH, CL, CYL and YCC wrote the article. All authors read and approved the final manuscript.
